# Survey about Intention to Engage in Specific Disaster Activities among Disaster Medical Assistance Team Members

**DOI:** 10.1017/S1049023X21001035

**Published:** 2021-12

**Authors:** Keita Iyama, Takeyasu Kakamu, Kazunori Yamashita, Jiro Shimada, Osamu Tasaki, Arifumi Hasegawa

**Affiliations:** 1. Department of Radiation Disaster Medicine, Fukushima Medical University, Fukushima, Japan; 2. Department of Hygiene and Preventive Medicine, Fukushima Medical University, Fukushima, Japan; 3. Acute and Critical Care Center, Nagasaki University Hospital, Nagasaki, Japan; 4. Futaba Emergency Medical Support Center, Fukushima Medical University, Fukushima, Japan

**Keywords:** disaster, emergency responders, hazard, human resources, intention

## Abstract

**Introduction:**

Different disaster activities should be performed smoothly. In relation to this, human resources for disaster activities must be secured. To achieve a stable supply of human resources, it is essential to improve the intentions of individuals responding to each type of disaster. However, the current intention of Disaster Medical Assistance Team (DMAT) members has not yet been assessed.

**Study Objective:**

To facilitate a smooth disaster response, this survey aimed to assess the intention to engage in each type of disaster activity among DMAT members.

**Methods:**

An anonymous web questionnaire survey was conducted. Japanese DMAT members in the nuclear disaster-affected area (Group A; n = 79) and the non-affected area (Group N; n = 99) were included in the analysis. The outcome was the answer to the following question: “Will you actively engage in activities during natural, human-made, and chemical (C), biological (B), radiological/nuclear (R/N), and explosive (E) (CBRNE) disasters?” Then, questionnaire responses were compared according to disaster type.

**Results:**

The intention to engage in C (50), B (47), R/N (58), and E (52) disasters was significantly lower than that in natural (82) and human-made (82) disasters (P <.001). The intention to engage in CBRNE disasters among younger participants (age ≤39 years) was significantly higher in Group A than in Group N. By contrast, the intention to engage in R/N disasters alone among older participants (age ≥40 years) was higher in Group A than in Group N. However, there was no difference between the two groups in terms of intention to engage in C, B, and E disasters. Moreover, the intention to engage in all disasters between younger and older participants in Group A did not differ. In Group N, older participants had a significantly higher intention to engage in B and R/N disasters.

**Conclusion:**

Experience with a specific type of calamity at a young age may improve intention to engage in not only disasters encountered, but also other types. In addition, the intention to engage in CBRNE disasters improved with age in the non-experienced population. To respond smoothly to specific disasters in the future, measures must be taken to improve the intention to engage in CBRNE disasters among DMAT members.

## Introduction

In patients with critical conditions, the initial response of the rapid response team or medical emergency team is the most important factor correlated with prognosis.^1–3^ In recent years, people have sustained injuries caused by different types of disasters, which can be classified as natural (ie, earthquakes), human-made (eg, transport accidents), and specific (ie, coronavirus disease 2019 [COVID-19] and chemical terrorism). Hence, the need to manage these disasters is increasing.^4^ Among them, chemical (C), biological (B), radiological (R), nuclear (N), and explosive (E) (CBRNE) disasters are considered specific. In such disasters, a rapid and smooth response is required to save the lives of patients. However, in the Fukushima Daiichi Nuclear Power Plant (FDNPP; Ōkuma, Fukushima, Japan) accident (2011), one of the most well-known radiological/nuclear (R/N) disasters, it was challenging to smoothly run disaster response activities at all times.^5^ Therefore, when providing medical treatment in areas with various hazards, measures should be taken in advance to facilitate disaster activities.

With consideration of factors that can prevent a smooth response to CBRNE disasters, the lack of human resources is a major concern. Disaster responders have a low intention to engage in specific disaster activities. Some surveys have shown that even individuals who are willing to respond to natural hazards avoid involvement in nuclear disasters or those involving communicable diseases due to anxiety and lack of knowledge.^6–8^ Hence, this is a major cause for the lack of human resources and is associated with difficulties in facilitating CBRNE disaster activities. A previous study revealed that factors such as self-confidence, incentives, and family understanding affect the intention of firefighters to engage in nuclear disaster activities.^9^ However, the current intention of all medical responders to participate in CBRNE disaster activities has not been fully elucidated.

In Japan, the Great Hanshin earthquake of 1995 has led to the development of a disaster medical system. Moreover, the Disaster Medical Assistance Team (DMAT), which responds to various disasters, has been established. The DMAT comprises physicians, nurses, and logisticians, as defined in the Basic Disaster Management Plan based on the Japan’s Disaster Countermeasures Basic Act.^10,11^ Japanese DMAT members can decide whether or not to respond when they receive dispatch requests. On the other hand, to date, the team plays a major role in large-scale disasters in Japan, and its members are the most important disaster medical responders in Japan. Therefore, a survey about the intention of DMAT members to engage in short-term CBRNE disaster activities must be conducted to facilitate a smooth response.

This study aimed to conduct a web questionnaire survey among DMAT members from two different areas (one with nuclear disaster experience and the other without). To smoothen each specific disaster response, the current intentions of DMAT members to engage in CBRNE disaster activities were examined. Moreover, future measures that can improve such intentions were evaluated.

## Methods

**Figure 1.**
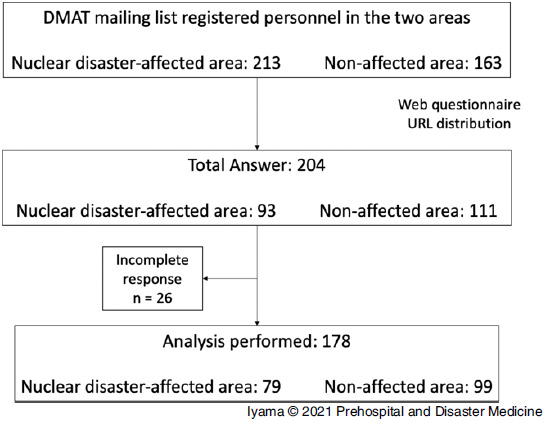
Flow Chart Showing the Selection of Participants. Note: The web URL of the questionnaire was sent via e-mail; addresses were in the two DMAT mailing lists. That is, one list was for a nuclear disaster-affected area and the other was for a non-affected area. In total, 204 members answered the questionnaire. After excluding 26 incomplete response data, 178 participants were finally included in the analysis. Abbreviation: DMAT, Disaster Medical Assistance Team.

This was a cross-sectional study. An anonymous web questionnaire survey was conducted from October 2020 through November 2020. The website URL of the questionnaire was distributed by research members via e-mail to DMAT members in the two different areas. That is, one was a nuclear disaster-affected area (Group A) and the other was a non-affected area (Group N). In total, 204 participants from both areas responded. However, only 178 provided complete responses (effective response rate: total 87.3%; Group A 84.9%; Group N 89.2%). These data were then included in the analysis (Figure 1). The sample size was estimated using the pwr.anova.test function of R 4.0.3 software (R Foundation for Statistical Computing; Vienna, Austria). The following three parameters were included: group size (k = 4), medium effect (f = 0.25), and power (0.8). The estimated sample size was 45 per group; therefore, the total size of the response group was determined to be 180. The questionnaire was used to collect information such as sex, age, occupation, family status, and experience in disaster activities. To validate the intention to engage in disaster activities (natural, human-made, CBRNE disasters), the following question was created: “Will you actively engage in response activities during a natural, human-made, or CBRNE disaster?” The participants were required to answer using the Engagement Intent Score (EIS), which indicates their agreement to the abovementioned question (0%-100%). Participants with an EIS of <50% were instructed to provide a free answer as to why they did not wish to engage.

**Figure 2.**
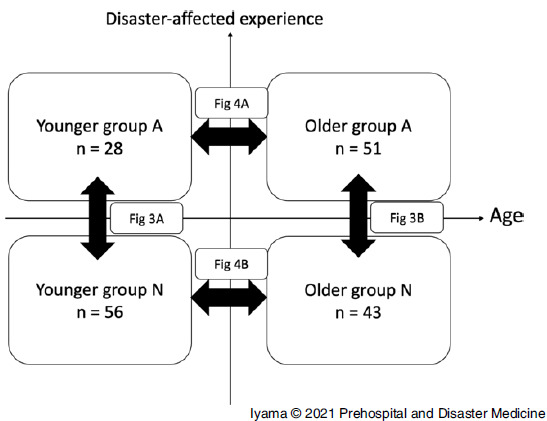
Diagrammatic Image Representation of a Stratified Comparison. Note: The horizontal axis indicates age and the vertical axis represents disaster experience. Each comparison in Figures 3A, 3B, 4A, and 4B is depicted with a black bidirectional arrow. Abbreviations: Group A, nuclear disaster-affected area; Group N, non-affected area.

The participants were divided into four groups according to age and area: younger Group A (≤39 years old; nuclear disaster-affected area), older Group A (≥40 years old; nuclear disaster-affected area), younger Group N (≤39 years old; non-affected area), and older Group N (≥40 years old; non-affected area; Figure 2). The reference age was set at 39 years as the mean age of DMAT members was 38.8 years, and a specific medical checkup is available for those aged >40 years in Japan.^12,13^ The background characteristics were compared between the four groups using the chi-squared test. Each EIS was presented as the mean and standard deviation (SD). The EIS between each disaster was compared with the analysis of variance and the Tukey-Kramer test for multiple comparisons. Sub-analysis was performed for male participants only. Further analyses were conducted according to age and area. The EIS was compared between the younger and older and nuclear disaster-affected and non-affected groups using the student’s t-test. All statistical analyses were performed using JMP 14 (SAS Institute Inc.; Cary, North Carolina USA), and a P value of .05 was considered statistically significant.

### Ethics Committee Approval

The ethics committee of Fukushima Medical University approved the study protocol (Fukushima, Japan; approval number: 2020-130).

## Results

The characteristics of younger participants in Group A (n = 28), younger participants in Group N (n = 56), older participants in Group A (n = 51), and older participants in Group N (n = 43) are depicted in Table 1. There were differences in terms of background characteristics between the four groups in terms of occupation and experience in natural disaster activities (Table 1). According to the primary outcome, the mean EIS for each type of disaster were as follows: natural, 82.2 (SD = 20.3); human-made, 81.7 (SD = 23.2); C, 50.0 (SD = 34.9); B, 47.4 (SD = 35.3); R/N, 57.6 (SD = 35.5); and E, 52.4 (SD = 36.1). After multiple comparisons, the proportion of natural and human-made disasters was significantly higher than that of C, B, R/N, and E disasters (all P values <.001). Furthermore, R/N disasters had a higher EIS than B disasters (P <.05; Table 2). In addition, a sub-analysis of only the male participants showed the same results (Supplemental Table 1; available online only).

**Table 1.** Characteristics of the Participants

	Younger Group A (n = 28)	Younger Group N (n = 56)	Older Group A (n = 51)	Older Group N (n = 43)	*P* Value
Sex, n (%)					
Female	6 (21.4)	17 (30.4)	18 (35.3)	9 (20.9)	.368
Male	22 (78.6)	39 (69.6)	33 (64.7)	34 (79.1)	
Age (years), n (%)					
20-29	6 (21.4)	8 (14.3)	−	−	.408^a^
30-39	22 (78.6)	48 (85.7)	−	−	
40-49	−	−	33 (64.7)	34 (79.1)	.125^b^
Over 50	−	−	18 (35.3)	9 (20.9)	
Occupation, n (%)					
Physician	3 (10.7)	8 (14.3)	17 (33.3)	13 (30.2)	.048
Nurse	7 (25.0)	27 (48.2)	20 (39.3)	15 (34.9)	
Administrative Staff	7 (25.0)	9 (16.1)	7 (13.7)	7 (16.3)	
Others	11 (39.3)	12 (21.4)	7 (13.7)	8 (18.6)	
Family, n (%)					
Without	6 (21.4)	16 (28.6)	11 (21.6)	10 (23.3)	.822
With	22 (78.6)	40 (71.4)	40 (78.4)	33 (76.7)	
Experience in Natural Disaster Activities, n (%)					
No	12 (42.9)	24 (42.9)	9 (17.6)	10 (23.3)	.012
Yes	16 (57.1)	32 (57.1)	42 (82.4)	33 (76.7)	
Experience in CBRNE Disaster Activities, n (%)					
No	27 (96.4)	53 (94.6)	45 (88.2)	39 (90.7)	.495
Yes	1 (3.6)	3 (5.4)	6 (11.8)	4 (9.3)	

Abbreviations: CBRNE, chemical, biological, radiological, nuclear, and explosive; Group A, nuclear disaster-affected area; Group N, non-affected area.

a Comparison between younger Group A and younger Group N.

b Comparison between older Group A and older Group N.

**Table 2.** Multiple Comparison of EIS between the Six Types of Disasters

No.	Disaster Type	Mean (SD) EIS	95% CI	*P* Value^a^				
				vs. 2	vs. 3	vs. 4	vs. 5	vs. 6
1	Natural	82.3 (SD = 20.3)	79.2-85.3	1.00	<.01	<.01	<.01	<.01
2	Human-Made	81.7 (SD = 23.2)	78.3-85.1	—	<.01	<.01	<.01	<.01
3	Chemical	50.0 (SD = 34.9)	44.8-55.1	—	—	.97	.21	.98
4	Biological	47.4 (SD = 35.3)	42.2-52.6	—	—	—	.03	.67
5	Radiological/Nuclear	57.6 (SD = 35.5)	52.3-62.8	—	—	—	—	.63
6	Explosive	52.4 (SD = 36.1)	47.0-57.7	—	—	—	—	—

Abbreviation: EIS, enga_gement intent score.

a P values <.05 were considered statistically significant.

**Figure 3.**
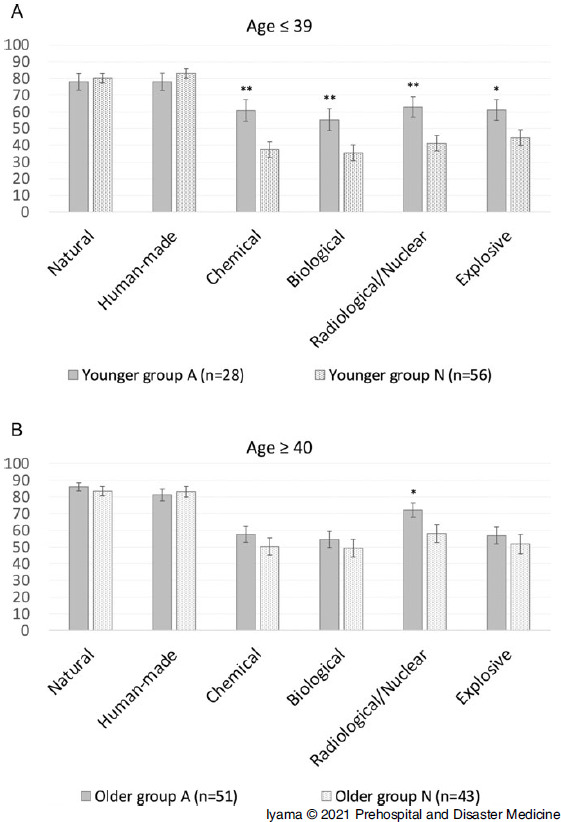
Comparison of Engagement Intent Score According to the Type of Disaster in Each Age Group. **A.** There was no significant difference between younger participants in Group A and Group N in terms of intention to engage in natural and human-made disasters. Group A had a significantly higher intention to engage in all CBRNE disaster activities. **B.** The score for radiological/nuclear disaster alone was significantly higher among older participants in Group A than in Group N. However, the results for other disasters, except radiological/nuclear ones, did not significantly differ between the two groups. Abbreviations: CBRNE, chemical, biological, radiological, nuclear, and explosive; Group A, nuclear disaster-affected area; Group N, non-affected area. * P <.05; ** P <.01.

**Figure 4.**
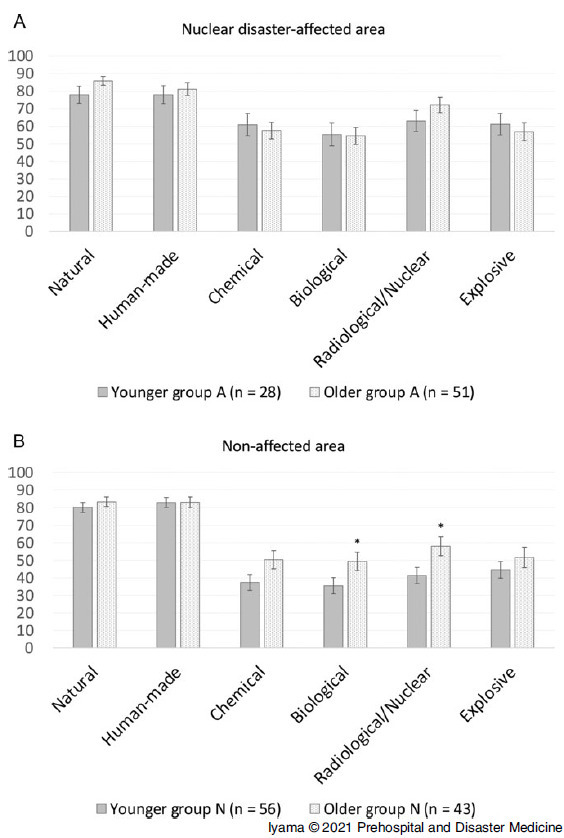
Comparison of Engagement Intent Score in Terms of the Type of Disasters in Each Group. **A.** There was no difference in the intention to engage in all types of disasters between younger and older participants in Group A. **B.** Older participants in Group N had a significantly higher intention to engage in biological and radiological/nuclear disaster activities. The same trend was observed for chemical disasters. However, the results did not significantly differ. Abbreviations: Group A, nuclear disaster-affected area; Group N, non-affected area. *P <.05.

Based on the intention to engage in various types of disasters, the EIS for all CBRNE disasters among younger participants was significantly higher in younger Group A than in younger Group N (C: 60.9 [SD = 30.8] versus 37.4 [SD = 32.3], P <.01; B: 55.3 [SD = 31.9] versus 35.5 [SD = 32.6], P <.01; R/N: 63.0 [SD = 31.9] versus 41.3 [SD = 35.0], P <.01; E: 61.1 [SD = 32.4] versus 44.6 [SD = 35.2], P <.05; Figure 3A and Table 3). Meanwhile, the EIS for R/N disasters alone among older participants was significantly higher in older Group A than in older Group N (72.1 [SD = 31.2] versus 58.0 [SD = 35.3]; P <.05), but those for other disasters (natural, human-made, C, B, and E) did not significantly differ between the two groups (Figure 3B and Table 3). According to age, there was no difference in the intention to engage in all types of disasters between younger and older participants in Group A (Figure 4A and Table 4). However, older participants in Group N had a significantly higher EIS for B (35.5 [SD = 32.6] versus 49.4 [SD = 35.7]; P <.05) and R/N (41.3 [SD = 35.0] versus 58.0 [SD = 35.3]; P <.05) disasters than younger participants in Group N (Figure 4B and Table 4).

**Table 3.** Comparison of EIS for Each Type of Disaster among the Same Age Group

Disaster Type	Younger Group A (n = 28)	Younger Group N (n = 56)	*P* Value^a^	Older Group A (n = 51)	Older Group N (n = 43)	*P* Value^a^
Natural	78.0 (SD = 25.9)	80.1 (SD = 20.9)	.68	85.9 (SD = 17.8)	83.4 (SD = 18.1)	.50
Human-Made	78.0 (SD = 27.1)	82.9 (SD = 21.2)	.36	81.2 (SD = 25.6)	83.1 (SD = 20.2)	.70
Chemical	60.9 (SD = 30.8)	37.4 (SD = 32.3)	<.01	57.6 (SD = 35.7)	50.3 (SD = 35.6)	.33
Biological	55.3 (SD = 31.9)	35.5 (SD = 32.6)	<.01	54.5 (SD = 37.3)	49.4 (SD = 35.7)	.50
Radiological/Nuclear	63.0 (SD = 31.9)	41.3 (SD = 35.0)	<.01	72.1 (SD = 31.2)	58.0 (SD = 35.3)	.04
Explosive	61.1 (SD = 32.4)	44.6 (SD = 35.2)	.04	56.8 (SD = 36.4)	51.6 (SD = 38.3)	.51

Abbreviation: EIS, engagement intent score.

a *P* values <.05 were considered statistically significant.

**Table 4.** Comparison of EIS for Each Type of Disaster among the Same Area

Disaster Type	Younger Group A (n = 28)	Older Group A (n = 51)	*P* Value^a^	Younger Group N (n = 56)	Older Group N (n = 43)	*P* Value^a^
Natural	78.0 (SD = 25.9)	85.9 (SD = 17.8)	.11	80.1 (SD = 20.9)	83.4 (SD = 18.1)	.42
Human-Made	78.0 (SD = 27.1)	81.2 (SD = 25.6)	.61	82.9 (SD = 21.2)	83.1 (SD = 20.2)	.97
Chemical	60.9 (SD = 30.8)	57.6 (SD = 35.7)	.68	37.4 (SD = 35.5)	50.3 (SD = 35.6)	.06
Biological	55.3 (SD = 31.9)	54.5 (SD = 37.3)	.93	35.5 (SD = 32.6)	49.4 (SD = 35.7)	.05
Radiological/Nuclear	63.0 (SD = 31.9)	72.1 (SD = 31.2)	.22	41.3 (SD = 35.0)	58.0 (SD = 35.3)	.02
Explosive	61.1 (SD = 32.4)	56.8 (SD = 36.4)	.60	44.6 (SD = 35.2)	51.6 (SD = 38.3)	.34

Abbreviation: EIS, engagement intent score.

a *P* values <.05 were considered statistically significant.

According to the free answers, the DMAT members were not willing to engage in natural and human-made disasters mainly due to safety and the thought of leaving their family members behind in case of injuries or death. Nevertheless, the main reasons why DMAT members were not willing to engage in CBRN disaster activities were lack of knowledge and skills along with the anxiety and fear attributable to the fact that the R or C agents cannot be visualized with the naked eye. Meanwhile, the reasons for not engaging in E disasters slightly differed. That is, several participants answered lack of security due to the risk of second or third explosion.

## Discussion

This study aimed to assess and elucidate the response provided by DMAT members to CBRNE disasters; this is considering the fact that an organized response is imperative in disaster management. To achieve the goal, this study initially focused on securing human resources. As a first step toward this goal, this study investigated the DMAT’s intention to engage in various types of disaster activities. Some questionnaire surveys asked about the intention to engage in disaster activities. However, most studies were based on a two-to-ten scale of responses to the questionnaire. To the best of the authors’ knowledge, this is the first survey about the intention of DMAT members to participate in a specific disaster activity, and the intention was scored on a scale of 100%. Using a continuous scale, the intention to engage in disaster activities could be accurately evaluated.

In previous studies, approximately 40% of medical personnel or firefighter-training students in a non-affected area^6,7^ and 56.3% in a nuclear disaster-affected area^9^ were willing to engage in R/N disaster activities. Based on these reports, disaster responders in a nuclear disaster-affected area had a higher intention to engage in R/N disasters, and this finding is understandable. With actual experience in helping individuals affected by a disaster, people can accept things as their own, and their interest in disaster activities increases. A greater interest leads to intention to take action.^14^ In this study, both age groups who experienced nuclear disasters had a higher EIS for R/N disasters, and the result was comparable to that of previous reports about firefighters in nuclear disaster areas. This finding indicated that experiences with disasters might have a positive influence on intention among not only firefighters, but also DMAT members.

As shown in Table 2, the EIS of CBRNE disasters was significantly lower than that of natural or human-made disasters. Moreover, the SD of the EIS in each CBRNE disaster was higher than that of natural or human-made disasters. This result indicates that the intention to engage in CBRNE disasters varies according to the member’s experience, knowledge, or skill; however, difference in the intention to engage in natural and human-made disasters appears to be less. According to the free answer, there was a trend for the reasons for not willing to engage in disaster activities. For natural and human-made disasters, the main reasons were safety and leaving family members behind in case of critical injuries or death. However, the main reasons for not willing to engage in CBRN disasters were lack of knowledge and skills, anxiety, and fear since these disasters cannot be prevented. The reasons for not willing to engage in E disasters slightly differed, and the most common reason was the lack of assurance regarding safety from the effects of second or third impact. Thus, DMAT members must have appropriate knowledge and skills to help improve their intention to engage in CBRNE disaster activities.

Biological disasters had the lowest EIS. This result may be attributed to the recent COVID-19 pandemic. That is, it is similar to a previous survey, which reported that there is a trend toward lower intention to engage in situations involving communicable diseases compared with R/N hazards.^7^ Japan experienced large C, R/N, or E disasters, such as the Tokyo subway sarin attack (1995), FDNPP accident (2011), and atomic bomb explosion during World War II (1945).^5,15,16^ By contrast, within the previous decades, there was no significant B disaster, except the COVID-19 pandemic. Moreover, lack of knowledge to disaster-related matters may result in an increase in the DMAT members’ perception of risks, which may consequently lead to avoidance in engaging to disaster activities.^17^ Based on these aspects, the fact of unknown might lead to a lack of disaster response image for this study population, and this might affect the result of low EIS in B disasters.

In the younger group (age: ≤39 years), the EIS was significantly higher in Group A than in Group N for all CBRNE disasters. It is easy to imagine that those who have experienced R/N disasters (Group A) have a higher EIS for R/N disasters. However, the result showed that they also had a higher EIS for C, B, and E disasters. In other words, the results indicated that the experience with R/N disasters had a positive impact on the intention to engage in other CBRNE disasters. Thus, an experience with one specific disaster may help develop adaptability or gain confidence to face other specific disasters in general, thereby improving one’s intention to engage to such disasters. In this study, the reason for the abovementioned phenomenon has not been validated. However, authors have considered the high-risk perception of young-aged Japanese population. A previous study has revealed that there is a relationship between behavior and risk perception.^18^ The latter is strongly influenced by not only experience, but also culture or nationality, and Japanese are known to have a higher-risk perception than other nationalities.^19,20^ Moreover, age affects risk perception.^19^ Individuals may perceive CBRNE disasters as similar based on experiences with a certain type of CBRNE disaster at a young age. Moreover, they can lower their risk perception for other CBRNE disasters. By contrast, the EIS for R/N disasters alone in older participants was significantly higher in Group A than in Group N, and there was no difference in the EIS for other CBRNE disasters between the two older groups. In the sub-analysis comparing the EIS of younger participants in Group A and older participants in Group N, there was no significant difference according to all types of disasters. Group A experienced R/N disasters at a young age, indicating that they acquired knowledge and experience at an early stage, which could have developed over a long period of time. As shown in Table 1, the background characteristics significantly differed in terms of occupation and natural disaster activity experience in each group. This study compared the EIS between the two groups according to each factor because it is impossible to exclude background characteristics if a simultaneous comparison of the four groups is performed.

Younger participants in Group N had a low EIS for CBRNE disasters. Thus, EIS increases with age and experience. Assessing what they have gained through aging or experience will help to identify the important points for interventions to elevate the EIS of individuals who engage in disaster activities in the future. There are several possible factors with consideration of age and experience. These include acquisition of knowledge and skills or interest. However, further research on specific measures should be conducted. By efficiently and accurately addressing these factors, the number of people who will engage in disaster activities will increase. Then, this will lead to a stable supply of human resources in the future.

## Limitations

The website URL of the questionnaire was sent to the registered e-mail addresses in the mailing list. However, whether all DMAT members have received the e-mail was not validated. Moreover, some people might have registered more than one e-mail address. Meanwhile, others might not have received the e-mail due to changes in address. Therefore, the actual collection rate was not verified. This survey was conducted on DMAT members from two areas only. Thus, DMAT members from other areas or individuals with other occupations that might involve engagement in various disasters were not included. Hence, further surveys must be conducted.

## Conclusion

Japanese DMAT members had a low intention to engage in CBRNE disaster activities compared with natural and human-made disaster activities. To respond smoothly to specific disasters, measures to efficiently improve the intention to engage in CBRNE disaster activities are required.

## Supplementary material

For supplementary material accompanying this paper visit https://doi.org/10.1017/S1049023X21001035. click here to view supplementary material
