# Supplementary material for: Survey about Intention to Engage in Specific Disaster Activities among Disaster Medical Assistance Team Members
Source: Prehosp Disaster Med. 2021 Oct 18;36(6):684–90. doi: 10.1017/S1049023X21001035 (PMC8607140; doi:10.1017/S1049023X21001035)
Supplement: Supplementary file 1 [file S1049023X21001035sup001.docx]

**Supplemental Table 1.** Multiple comparison of engagement intent score between six disasters for male respondents to our survey

| **No.** | **Disaster type** | **Mean (SD) EIS** | **95% CI** | ***P* Value** | | | | |
| --- | --- | --- | --- | --- | --- | --- | --- | --- |
|  |  |  |  | **vs. 2** | **vs. 3** | **vs. 4** | **vs. 5** | **vs. 6** |
| 1 | Natural | 82.3 (20.3) | 79.2–85.3 | 1.00 | <.01 | <.01 | <.01 | <.01 |
| 2 | Human-made | 81.7 (23.2) | 78.3–85.1 | — | <.01 | <.01 | <.01 | <.01 |
| 3 | Chemical | 50.0 (34.9) | 44.8–55.1 | — | — | 0.98 | 0.42 | 0.99 |
| 4 | Biological | 47.4 (35.3) | 42.2–52.6 | — | — | — | 0.11 | 0.81 |
| 5 | Radiological/Nuclear | 57.6 (35.5) | 52.3–62.8 | — | — | — | — | 0.78 |
| 6 | Explosive | 52.4 (36.1) | 47.0–57.7 | — | — | — | — | — |

CI, confidence interval; EIS, engagement intent score; SD, standard deviation.

*p*-values <0.05 were considered statistically significant.
